# 

**Published:** 1917-03-24

**Authors:** 


### CONTENTS.

PAGE

**Insane Soldiers and the Law** ... ... ... 487

**The Medical Profession and National Service** 488

**Hospital and Institutional News** ... ... ... 489

The Duchess of Connaught—The Hostel Committee for Victims of Nerve Strain—Mr. Peter Reid's Bequests—Another Large Estate for Institutions—Tuberculosis in the Army—Attendance at Annual Meetings—Removal of Children from Hospital Construed as Parental Cruelty—The Folly of Closing a Cottage Hospital To-day—A "Vanishing" Class in York—Sea Tales and Food Rations—The Public Performance of "Damaged Goods"—A Retrograde Step at Merthyr—The Maintenance of Sanatorium Beds—The Unprovided Consumptive in Wales—An Extraordinary Decision—The Centenary of a Welsh Hospital—Catheterisation of Male Patients.

**The Psychology of Fear** ... ... ... ... 493

The Effects of Panic Fear in Wartime.

**Flavine** ... ... ... ... ... ... ... 495

The Ideal Antiseptic.

**The War and our Supply of Drugs** ... ... 497

**Hospital Architecture and Construction** ... 498

The Royal Gwent Hospital, Newport, Mon.

PAGE

**A Chapter in Hospital History** ... ... ... 499

A Century of Rebuilding at Leeds.

**Parliament and the Profession** ... ... 500

**Hospital and Institutional Results** ... ... 500

**The Matrons' and Sisters' Department** ... 501

The Problem of Poor-Law Infirmaries: The Workhouse Influence.

Round the Hospitals.

Expected Developments in Ireland—Membership of State Registration Society—M.P.s and College Members *in Forma Pauperis?* "The Only Professional Nursing Journal"—State Registrationists in Disagreement—A Mountain in Labour.

**Some War Problems in Food** ... ... ... 504

II. Meat Rations for the Staff.

**From Across the Seas** ... ... ... ... 505

**Matrons' and Sisters' Appointments** ... ... 506

**Annual Meetings** ... ... ... ... ... 506

**The Institutional Worker** ... ... (Suppl.) 1

The Service of Patients' Dinners.

Questions for March.

Enquiries and Answers.

The Sick and in Need.

